# A Trivalent Virus-Like Particle Vaccine Elicits Protective Immune Responses against Seasonal Influenza Strains in Mice and Ferrets

**DOI:** 10.1371/journal.pone.0006032

**Published:** 2009-06-24

**Authors:** Ted M. Ross, Kutub Mahmood, Corey J. Crevar, Kirsten Schneider-Ohrum, Penny M. Heaton, Rick A. Bright

**Affiliations:** 1 Center for Vaccine Research, University of Pittsburgh, Pittsburgh, Pennsylvania, United States of America; 2 Department of Microbiology and Molecular Genetics, University of Pittsburgh, Pittsburgh, Pennsylvania, United States of America; 3 Novavax, Inc., Rockville, Maryland, United States of America; Institute of Molecular and Cell Biology, Singapore

## Abstract

There is need for improved human influenza vaccines, particularly for older adults who are at greatest risk for severe disease, as well as to address the continuous antigenic drift within circulating human subtypes of influenza virus. We have engineered an influenza virus-like particle (VLP) as a new generation vaccine candidate purified from the supernatants of Sf9 insect cells following infection by recombinant baculoviruses to express three influenza virus proteins, hemagglutinin (HA), neuraminidase (NA), and matrix 1 (M1). In this study, a seasonal trivalent VLP vaccine (TVV) formulation, composed of influenza A H1N1 and H3N2 and influenza B VLPs, was evaluated in mice and ferrets for the ability to elicit antigen-specific immune responses. Animals vaccinated with the TVV formulation had hemagglutination-inhibition (HAI) antibody titers against all three homologous influenza virus strains, as well as HAI antibodies against a panel of heterologous influenza viruses. HAI titers elicited by the TVV were statistically similar to HAI titers elicited in animals vaccinated with the corresponding monovalent VLP. Mice vaccinated with the TVV had higher level of influenza specific CD8+ T cell responses than a commercial trivalent inactivated vaccine (TIV). Ferrets vaccinated with the highest dose of the VLP vaccine and then challenged with the homologous H3N2 virus had the lowest titers of replicating virus in nasal washes and showed no signs of disease. Overall, a trivalent VLP vaccine elicits a broad array of immunity and can protect against influenza virus challenge.

## Introduction

The influenza A virus, a member of the *Orthomyxoviridae* family, is an enveloped segmented, negative-strand RNA virus with a genome consisting of eight individual genes that encode at least ten proteins [1]. Influenza A viruses are further subdivided by antigenic characterization of the hemagglutinin (HA) and neuraminidase (NA) surface glycoproteins. Currently, there are 16 identified HA and 9 NA subtypes [2]. Waterfowl, such as ducks and geese, serve as a natural reservoir for all known subtypes of influenza A virus [3]. Annually, human outbreaks of influenza types A subtypes, currently H1N1 and H3N2 and influenza B are responsible for substantial morbidity and mortality in humans [4]. High-risk groups, such as elderly, infants, and immunocompromised individuals are most susceptible to infection and severe disease.

Prevention is the most effective method of reducing transmission of influenza [5] and protection is primarily mediated by antibodies to the HA and NA (see reviews [6], [7]. The HA is responsible for attachment of the virus to human epithelial cells that line the upper respiratory tract as well as fusion of the viral and cellular lipid membranes during initial stages of infection. The NA has enzymatic properties that are associated with the release of nascent virions from cell membranes following viral replication [8]. Annual influenza epidemic and periodic pandemic outbreaks result from continuous antigenic changes within HA and NA proteins, known as antigenic drift and shift. During antigenic drift, HA and NA surface antigens undergo progressive amino acid substitutions that can result in evasion of the previously acquired immunity. Therefore, currently licensed influenza vaccines can vary widely in their level of efficacy from year to year due to selection of a vaccine strain does not sufficiently match the circulating virus strain within a population. Surveillance within avian and human populations is a cornerstone of the World Health Organization's influenza surveillance network which each year identifies newly emerging influenza strains circulating among humans throughout the northern and southern hemisphere and convenes bi-annually to recommend new influenza strains believed to be suitable for subsequent annual vaccine manufacture based on epidemiological and antigenic considerations and their anticipated prevalence during the coming season [9].

Traditionally, inactivated whole, split or purified influenza A and B virus vaccines are prepared by culturing live virus in embryonated chicken eggs. There are limitations to relying on an egg-based manufacturing system including egg allergies in a small percentage of the population as well as potential issues with egg supply for surge capacity or potential depletion of egg supply due to avian influenza outbreaks. Alternative influenza vaccine manufacturing platforms based upon scalable and recombinant approaches could therefore be of great public benefit.

Recently, we have described the development of influenza A H3N2, H5N1, and H9N2 VLP vaccine candidates that were comprised of three influenza virus structural proteins, HA, NA, and M1 and expressed from insect cells [10]–[13] and others have described similar VLPs based upon a lentiviral core [14]. This new generation vaccine candidate has potential advantages over current egg-based methods, particularly for immunogenicity and high-yielding, inexpensive production. In this study, VLP vaccines were constructed for a seasonal trivalent vaccine using isolates from influenza A H1N1 (A/New Caledonia/20/1999), H3N2 (A/New York/55/2004), and influenza B (B/Shanghai/367/2002) to match strains recommended for the commercially marketed seasonal 2005–2006 Northern Hemisphere vaccine formulation. This investigational seasonal influenza vaccine is composed of non-infectious, non-replicating VLPs that exhibit functional HA and NA properties. These vaccines were tested in both mice and ferrets for the induction of immune responses that correlate with protection and these elicited immune responses were compared to immune responses elicited from the corresponding monovalent VLP in the same animal study. In addition, immune responses elicited in mice by the TVV and by the commercial TIV were compared.

## Materials and Methods

### Cloning of HA, NA, and M1 genes and the generation of recombinant baculoviruses

Abbreviations for the H3N2 viral isolates or proteins used in this study were: A/Brisbane/10/2007 (Bris/10/07), A/Wisconsin/67/2005 (Wisc/05), A/New York/55/2004 (NY/04), A/Wyoming/4/2003 (Wyo/03), Fujian/411/2002 (Fuj/02), A/Panama/2007/99 (Pan/99), A/Aichi/2/1968 (Aichi/68). Abbreviations for the H1N1 viral isolates or proteins used in this study were: A/Brisbane/59/2007 (Bris/59/07), A/Solomon Island/3/2006 (SI/06), A/New Caledonia/20/1999 (NC/99), A/Puerto Rico/8/1934 (PR/8/34). Abbreviations for influenza B viruses or proteins used in this study were B/Florida/4/2007 (B/FL/07), B/Malaysia/2506/2004 (B/May/04), B/Shanghai/361/2002 (B/Shang/02), B/Sichuan/379/99 (B/Sich/99). Influenza VLPs representing NY/04, NC/99, B/Shang/02 were constructed and purified from a baculovirus/insect cell expression system as previously described [11], [13]. Particle production was analyzed by sucrose gradient ultracentrifugation and chromatography followed by Western blot as described [11].

### Comparative antigens and controls

Wyo/03 virus was inactivated using 0.1% Formalin (Center for Biological Evaluation and Research (CBER), Food and Drug Administration (FDA), Rockville, MD, USA) and rHA (Lot # 45-04028) derived from Wyo/03 virus was purified from the supernatants of Sf9 insect cells following baculovirus infection (Protein Sciences Corp., Meriden, CT, USA). The previously described the HIV-1 VLP [15], expressed from the HIV-1_NL4-3_ Gag plasmid was used as a negative viral particle control in this study. 2007–2008 commercial trivalent split FLUARIX (GlaxoSmithKline, Research Triangle Park, NC, USA) vaccine was used as a comparator control.

### Animals and vaccinations

BALB/c mice (*Mus musculis*, females, 6–8 weeks) were purchased from Harlan Sprague Dawley, (Indianapolis, IN, USA) and housed in microisolator units and allowed free access to food and water and were cared for under USDA guidelines for laboratory animals. Mice (12–21 mice per group) were vaccinated with purified VLPs (3 µg, 0.6 µg, or 0.12 µg), based upon HA content from a SRID potency assay, via intramuscular injection at week 0 and then boosted with the same dose at week 3. A subset of mice was vaccinated with the FLUARIX TIV vaccine (TIV) at a 0.6 µg HA dose to match the dose of VLP vaccine. Blood was collected from anesthetized mice via the orbit and transferred to a microfuge tube. Tubes were centrifuged and sera was removed and frozen at −80±5°C. All procedures were in accordance with the NRC Guide for the Care and Use of Laboratory Animals, the Animal Welfare Act, and the CDC/NIH Biosafety in Microbiological and Biomedical Laboratories.

Fitch ferrets (*Mustela putorius furo*, male, 6–12-months of age), influenza naïve and descented were purchased from Marshall Farms (Sayre, PA, USA). Ferrets were pair housed in stainless steel cages (Shor-line, Kansas City, KS, USA) containing Sani-chips Laboratory Animal Bedding (P.J. Murphy Forest Products, Montville, NJ, USA). Ferrets were provided with Teklad Global Ferret Diet (Harlan Teklad, Madison, WI, USA) and fresh water *ad libitum*. Influenza VLPs were diluted in PBS, pH 7.2 to achieve final concentration. Ferrets (n = 6) were vaccinated with one of three doses (15 µg, 3 µg, 0.6 µg) of purified VLPs (NY/04, NC/99, and B/Shang/02 in monovalent or trivalent formulations) in the left hind leg muscle in a volume of 0.5 ml. Vaccines were stored at 4°C prior to use. Control animals were mock vaccinated with an HIV-1 VLP (ADA strain) as a negative control. Animals were monitored for weight loss, temperature, loss of activity, nasal discharge, sneezing and diarrhea weekly during the vaccination regimen and each day during viral challenge. Prior to vaccinations, animals were confirmed by HAI assay to be seronegative for circulating influenza A (H1N1 and H3N2) and influenza B viruses.

Blood was collected from anesthetized ferrets via the anterior vena cava. Blood was transferred to a tube containing a serum separator and clot activator and allowed to clot at room temperature. Tubes were centrifuged and sera was removed and frozen at −80±5°C. All procedures were in accordance with the NRC Guide for the Care and Use of Laboratory Animals, the Animal Welfare Act, and the CDC/NIH Biosafety in Microbiological and Biomedical Laboratories.

### HAI antibody levels in sera

The hemagglutination inhibition (HAI) assay was adapted from the CDC laboratory-based influenza surveillance manual (3). To inactivate non-specific inhibitors, sera were treated with receptor destroying enzyme (RDE) prior to being tested (6, 7). Briefly, three parts RDE was added to one part sera and incubated overnight at 37°C. RDE was inactivated by incubation at 56°C for ∼30 min and then six parts of saline were added, creating a 1∶8 dilution of serum. RDE-treated sera were two-fold serially diluted in v-bottom microtiter plates. An equal volume of virus, adjusted to approximately 4 HAU/25 µl was added to each well. The plates were covered and incubated at room temperature for 15 min followed by the addition of 0.5% turkey erythrocytes (TRBC) (Lampire Biologicals, Pipersville, PA, USA). The TRBCs were adjusted with PBS to achieve a 0.5% vol/vol suspension. The cells were stored at 4°C and used within 72 hours of preparation (2). The plates were mixed by agitation, covered, and the TRBC were allowed to settle for 30 min at room temperature. The HAI titer was determined by the reciprocal dilution of the last well which contained non-agglutinated TRBC. Positive and negative serum controls were included for each plate. All mice and ferrets were negative (HAI≤10) for pre-existing antibodies to currently circulating human influenza viruses prior to vaccination (1).

### ELISPOT assays

Spleens and lung tissue were harvested from vaccinated mice at day 6 post-challenge (day 41 of the study) and collected cells were isolated for ELISPOT assays, as previously described [16], [17]. Whole lungs or spleens were removed and forced into a single cell suspension using a cell strainer (BD Biosciences, Bedford, MA, USA) in a total volume of 4 ml. Red blood cells were then lysed using 5 ml of ACK buffer (0.15 M NH_4_Cl, 10 mM KHCO_3_ and 0.1 mM Na_2_EDTA). The lung and spleen cells were then resuspended in 1 ml and 3 ml, respectively, of RPMI medium with 10% fetal bovine serum (cRPMI). Cell viability was determined by trypan blue exclusion staining.

Briefly, splenocytes were depleted of erythrocytes by treatment with ammonium chloride (0.1 M, pH 7.4). Following thorough washing with PBS, cells were resuspended in RPMI medium with 10% fetal bovine serum (cRPMI). Cell viability was determined by trypan blue exclusion staining. The number of anti-HA or anti-M1 specific murine IFN-γ (mIFN-γ) secreting splenocytes was determined by enzyme-linked immunospot (ELISPOT) assay (R & D Systems, Minneapolis, MN, USA). Briefly, pre-coated anti-mIFN-γ plates were incubated (25°C for 2 h) with cRPMI (200 µl) and then were incubated with splenocytes or lung cells (5×10^5^/well) isolated from vaccinated mice. Splenocytes or lung cells were stimulated (48 h) with peptides (BEI Research Resources Repository, Manassas, VA, USA). For HA, six pools of peptides (15mers overlapping by 11 amino acids) and for M1, four peptides pools (15mers overlapping by 11 amino acids) from the A/New Calendonia/20/1999 were used to stimulate cells. Additional wells of cells were stimulated with PMA (50 ng)/ionomycin (500 ng) or were mock stimulated. In addition, IL-2 was added to all wells (10 units/ml). Plates were washed with PBS-Tween (3X) and were and were incubated overnight at 4C with anti-IFN-γ antibody. The plates were washed and then incubated (25°C for 2 h) with strepavidin conjugated to alkaline phosphatase. Following extensive washing, cytokine/antibody complexes were incubated (25°C for 1 h) with stable BCIP/NBT chromagen. The plates were rinsed with dH_2_O and air dried (25°C for 2 h). Spots were counted by an ImmunoSpot ELISPOT reader (Cellular Technology Ltd., Cleveland, OH, USA).

### Flow cytometry

A multi-parameter flow cytometry assay combining MHC class I pentamer with intracellular cytokine staining was employed to detect influenza specific CD8^+^ T cells. The CD8^+^ T cell responses to nucleoprotein (NP) 147 (TYQRTRALV) are dominant followed by hemagglutinin (HA) 533 (IYSTVASSL) responses in influenza-virus-infected BALB/c mice. HA_533_ was originally described for the HA of PR8 (H1N1) [18]. This epitope is conserved in H1N1 viruses. Lung lymphocytes and splenocytes of infected mice were stimulated for 5 h with 1 mg/ml of HA_533_, NP_147_, or an ovalbumin Ova_257_ control peptide in the presence of the Golgi-blocking agents brefeldin A and monensin. The cells were washed with FACS buffer (PBS, 1% FBS, 0.1% sodium azide) and were blocked with anti-CD16/CD32 mouse Fc receptor block (BD Biosciences, San Jose, CA, USA), followed by staining with a murine MHC-I encoded allele K^d^-specific pentamer for the immunodominant HA _533_ epitope or NP_147_ epitope (ProImmune, Oxford, UK) conjugated with phycoerythrin (PE). Lymphocytes were then stained with anti-CD8 antibodies conjugated with Pacific Blue and anti-CD19 antibodies conjugated with APC-Cy7 (BD Biosciences, San Jose, CA, USA). The cells are then incubated with a viability dye (Molecular Probes, Invitrogen, Eugene, OR, USA). Once the surface staining is complete the cells are fixed and permeabilized with BD Cytofix/Cytoperm solution (BD Biosciences, San Jose, CA, USA). The cells were washed in Perm/Wash buffer (BD Biosciences, San Jose, CA, USA) and the intracellular antibodies were added in Perm/Wash buffer. Cells were stained intracellular with FITC conjugated anti-IL-2 (eBioscience, San Diego, CA, USA), PE-Cy7 conjugated anti-IFN-γ (BD Biosciences, San Jose, CA, USA) and Alexa Fluor 488® conjugated anti-TNFα (eBiosciences, San Diego, CA, USA). Cells were fixed in 1% formalin/PBS and data acquired using a LSRII flow cytometer (BD Biosciences, San Jose, CA, USA). The data were analyzed with FlowJo software (Treestar, Ashlend, OR, USA).

### Challenge with influenza virus

Mice were challenged intranasally with 7.25×10^5^ pfu of mouse-adapted (ma) NC/99 (H1N1). Mice were monitored daily for morbidity and body weights were recorded each day. Mice that lost greater than 20% of body weight were euthanized. The ability of each vaccine to protect against challenge was compared to separate groups of mock vaccinated control mice that were challenged with the virus.

Vaccinated ferrets were challenged intranasally with 5.0×10^5^ pfu of influenza NY/04 virus (H3N2). Nasal wash samples were taken from ferrets on days 1, 3, and 5 post-challenge and influenza virus was titrated in MDCK cells to determine virus shedding in upper respiratory tissues [19]. Samples were collected, snap frozen at −70°C and later thawed, homogenized in 1 ml of cold PBS and pelleted by centrifugation. Clarified homogenates were titrated in MDCK cells for virus infectivity from initial dilutions of 1∶10. The limit of virus detection was 1×10^2^ pfu/ml.

### Statistical analysis

Statistical analyses were performed using the Student's *t*-test. Samples from VLP-vaccinated mice and ferrets were compared to TIV-vaccinated or mock vaccinated mice and significance was considered at a *p*-value *<*0.05.

## Results

### Serum HAI antibody measurements following VLP immunizations

**Figure 1:**
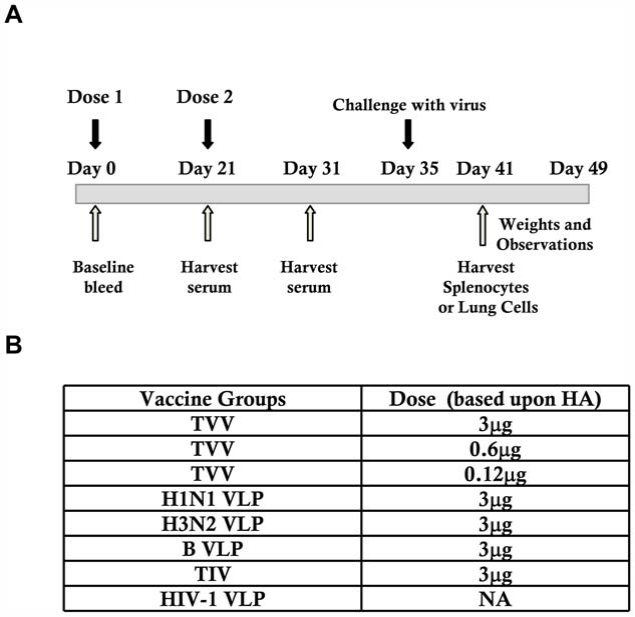
Antibodies elicited by vaccination. Mice (n = 8) were vaccinated via intramuscular injection at weeks 0 and 3 with VLPs representing a H1N1 virus (NC/99), a H3N2 VLP (NY/04), or a B VLP (B/Shang/02) individually (3 ug) or in a trivalent mixture at one of three doses (3 µg, 600 ng, or 120 ng). A) Schematic of the vaccine regimen. B) Vaccines and doses administered.

**Figure 2:**
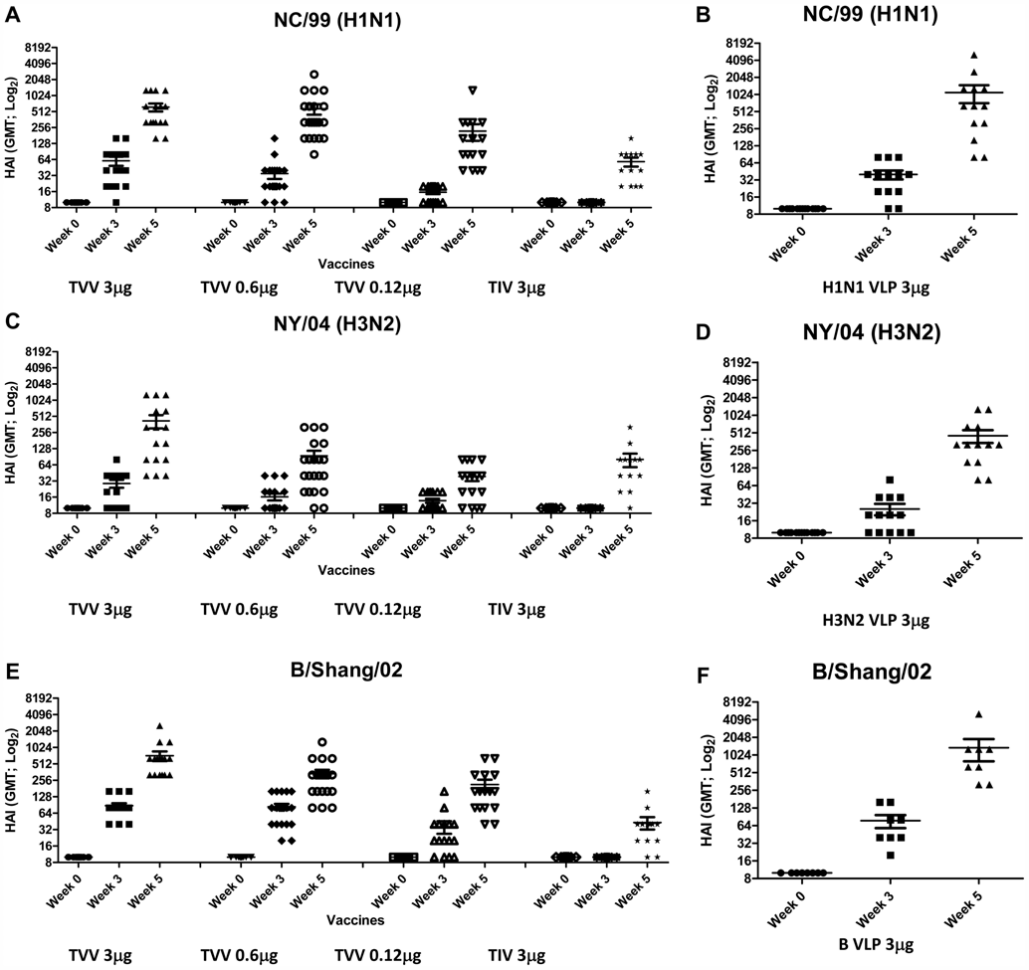
Hemaggutination-inhibition (HAI) titers. Week 0, 3, and 5 serum HAI antibody responses were assessed against H1N1 (NC/99), H3N2 (NY/04), B, (B/Shang/02) viruses. Bars indicate geometric mean titer (GMT) +/− SEM. Mice vaccinated with one of three doses of the TVV (A, C, E) or each VLP individually; H1N1 VLP (B), H3N2 VLP (D), B VLP (F).

Individual recombinant baculoviruses were used to infect Sf9 insect cells that subsequently expressed HA, NA, and M1 proteins from either NC/99 (H1N1), NY/04 (H3N2), or B/Shang/02 strains of influenza virus to spontaneously form VLPs that were then purified as previously described [11]. Immunogenicity of VLPs was examined in BALB/c mice (6–8 weeks) inoculated by intramuscular injection (i.m.) at 0 and 3 weeks with purified monovalent (3 µg) or a trivalent mixtures (3 µg, 0.6 µg, 0.12 µg) of the antigen (Fig. 1). Post-vaccination antisera were evaluated for the ability to prevent virus-induced agglutination of turkey RBCs (Fig. 2). These results were compared to mice vaccinated with the commercial split TIV (3 µg). At week 3, following the priming dose, 75% of the mice vaccinated with 3 µg of the TVV had measurable titers of HAI antibodies (≥1∶40) against the NC/99 (Fig. 2A) while forty-seven percent had HAI titers against NY/04 (Fig. 2C) and 100% of the mice had HAI titers against B/Shang/02 (Fig. 2E). At week 5, following two vaccinations, all mice immunized with the TVV at 3 µg had HAI titers against NC/99 (GMT 640), NY/04 (GMT 423) and B/Shang/02 (GMT 640). Mice that were vaccinated with a 3 µg dose of each monovalent VLP vaccine had HAI titers to homologous strains that were statistically similar to the corresponding HAI titer elicited by the TVV at the same dose (Fig. 2B, 2D, and 2F). All mice vaccinated with monovalent VLPs had HAI titers ≥1∶40. There was little, if any, HAI cross-reactivity against viruses from non-corresponding subtypes (data not shown). In contrast, 70–75% of the mice vaccinated with TIV had HAI titers ≥1∶40 to one of the three homologous viruses (Fig. 2). In addition, mice vaccinated with TVV had statistically higher HAI GMT compared to mice vaccinated with TIV, regardless of influenza strain tested. For NC/99 and B/Shang/02, HAI titers elicited in mice vaccinated with 0.12 µg of TVV were statistically higher than mice vaccinated with 3 µg of TIV. Therefore, in general, the TVV was inducing higher HAI titers than the TIV, with broader cross-protection.

### Influenza virus challenge of TVV and TIV vaccinated mice

**Figure 3:**
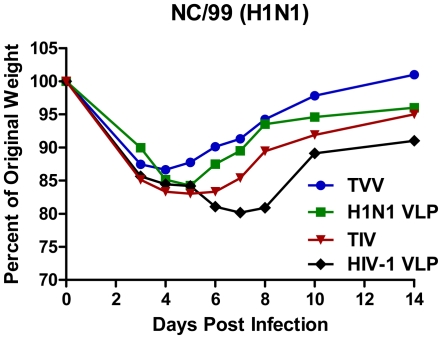
Influenza virus challenge. At week 5, mock vaccinated mice or mice vaccinated with vaccines (3 µg) were challenged intranasally with the mouse-adapted influenza ma-NC/99 virus (2.6×10^5^ pfu). Mice were monitored daily for weight loss, activity, and survival. Body weight is plotted as percentage of the average initial weight. Mice that lost greater than 20% body weight were sacrificed.

Mice vaccinated with H1N1 VLPs, the TVV, or the TIV were challenged with a mouse-adapted NC/99 (ms-NC/99) (Fig. 3). We had a limited number of mouse-adapted viruses to choose for these studies. The NC/99 virus matched the H1N1 component in these vaccines. HIV-1 VLP vaccinated mice challenged with virus showed physical signs of infection (ruffled fur, dyspnea, lethargy) and they lost 15–20% of their original body weight between days 4–6 post-challenge (Fig. 3). Sixty percent of these mice died by day 8 post-infection and the remaining mice recovered. Surprisingly, mice vaccinated with the H1N1 VLP, TVV, or TIV and then challenged with ma-NC/99 virus lost a similar amount of weight as the mock control animals, but appeared to recover more quickly (Fig. 3A). Even though, all vaccinated and mock-vaccinated mice had similar high viral lung titers at day 3 post-challenge (Table 1), all the mice vaccinated with the H1N1 VLP, TVV, or TIV survived challenge. As a control, mice were vaccinated with a mouse-adapted B influenza virus (ma-B/Sich/02) and all the monovalent B, TVV and TIV mice showed no signs of weight loss and had no outward signs of disease, had little or no B/Sich/02 virus detected in the lungs, whereas HIV-1 VLP vaccinated died by day 6 post-infection (data now shown).

**Table 1:** Virus titers in lungs of mice challenged with mouse-adapted H1N1.

Vaccine^a^	Virus titer (pfu/ml) in lungs
	NC/99 (H1N1)
TVV	2.3×10e+5
H1N1 VLP	6.3×10e+4
TIV	1.0×10e+5
HIV-1 VLP	9.0×10e+5

a Vaccine administered at weeks 0 and 3.

### Cell-mediated immunity elicited by VLP vaccines

**Figure 4:**
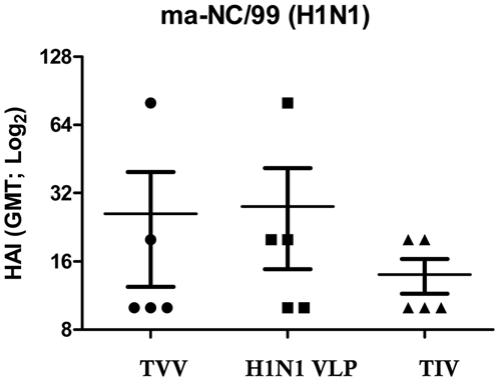
HAI titers against mouse-adapted viruses. Week 5 serum HAI antibody responses were assessed against ma-NC/99 virus. Bars indicate geometric mean titer (GMT) +/− SEM.

It was an unexpected finding that vaccinated mice, subsequently challenged with the mouse-adapted NC/99 virus, lost ∼15% of their original body weight, since all the vaccines contained NC/99 HA and they both elicited high HAI titers against NC/99 virus (Fig. 2). Therefore, we tested the antisera against the mouse-adapted NC/99 virus used for challenge. Interestingly, there was very low HAI activity against the mouse-adapted NC/99 virus (Fig. 4A) and only 20% of the mice had titers ≥1∶40, whereas all the mice had high titers against the wild-type human NC/99 virus (Fig. 2A). The vaccines contained the human sequences. The virus was mouse-adapted. The HAI titers elicited by the TVV and TIV were compared to the human strains. Then, as described on page 10, we verified if the vaccines that contained the human NC/99 HA elicited HAI antibodies that recognized mouse-adapted NC/99 HA. The number of differences in the mouse adapted HA and the human HA are 12.

**Figure 5:**
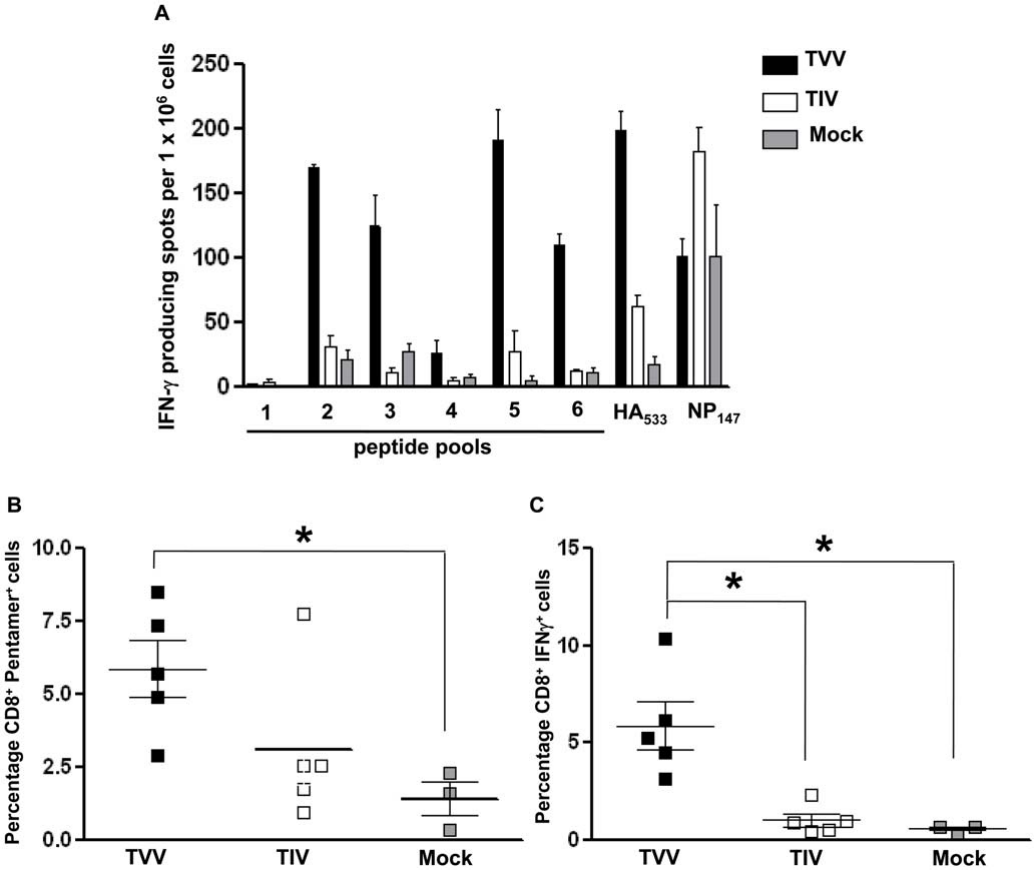
T cell responses. A. IFN-gamma ELISPOT. Splenocytes were collected on day 6 post-infection with mouse-adapted NC/99 (H1N1). B. Intracellular cytokine staining of CD8+ HA_533_-specific pentamer positive lung lymphocytes collected on day 6 post challenge from mice immunized with TVV, TIV or non-immunized (Mock). C. Percentage of the positive cells in panel B expressing IFN-gamma detected by intracellular cytokine staining. *represents p<0.05.

Viral infection and subsequent weight loss in vaccinated mice challenged with ma-NC/99 virus provided an opportunity to examine the cellular responses in these mice post-challenge, since vaccinated mice did appear to recover more quickly from NC/99 virus infection than unvaccinated mice (Fig. 3A). Splenocytes and lung cells were collected at day 6 post-challenge from mice vaccinated with TVV, TIV, or mock control animals. At 6 day post infection, the T cell response was low, unless there was preexisting memory allowing for a quicker recall response. Splenocytes were stimulated *in vitro* in an IFNγ-ELISPOT assay with 6 pools of overlapping peptides representing the HA protein or with peptides stimulation CD8^+^ T cells specific for the immunodominant epitopes HA_533_ (IYSTVASSL) or NP_147_ (TYQRTRALV). In both H1N1 vaccine strains in the TVV and TIV, the HA_533_ epitope is conserved in the HA protein and therefore allows for direct comparison of the CD8^+^ T cell responses. As expected, mice vaccinated with the TIV had high HAI titers against the three homologous viruses in the TIV (data not shown). In contrast, HA-specific cellular responses were elicited by the TVV, but not the TIV (Fig. 5). T cell responses were directed against epitopes throughout the HA (pools 2, 3, 5, and 6) following vaccination with the TVV. In addition, approximately 3 times the number of HA_533_ specific CD8^+^ T cells was detected in mice vaccinated with the TVV compared to TIV-vaccinated mice (Fig. 5). As expected, CD8^+^ T cells specific for the NP_147_ epitope were only detected above background in TIV vaccinated mice, since the VLP vaccines did not contain NP. There were no differences in the number of low cellular responses detected against M1 antigen (data not shown). In mice vaccinated with VLPs, there were approximately twice as many HA_533_-pentamer positive CD8^+^ T cells in the lung compared to TIV vaccinated mice, as shown by flow cytometry (Fig. 5B). Intracellular cytokine staining for IFN-γ revealed that approximately 6% of CD8^+^ T cells in the lungs of VLP vaccinated mice produce IFNγ after *in vitro* stimulation with the HA_533_ peptide (Fig. 5C), which was significantly higher than in TIV-immunized mice (1%). As expected, control mice had few HA-specific responses 6 days post-challenge.

### Influenza challenge of VLP vaccinated ferrets

**Figure 6:**
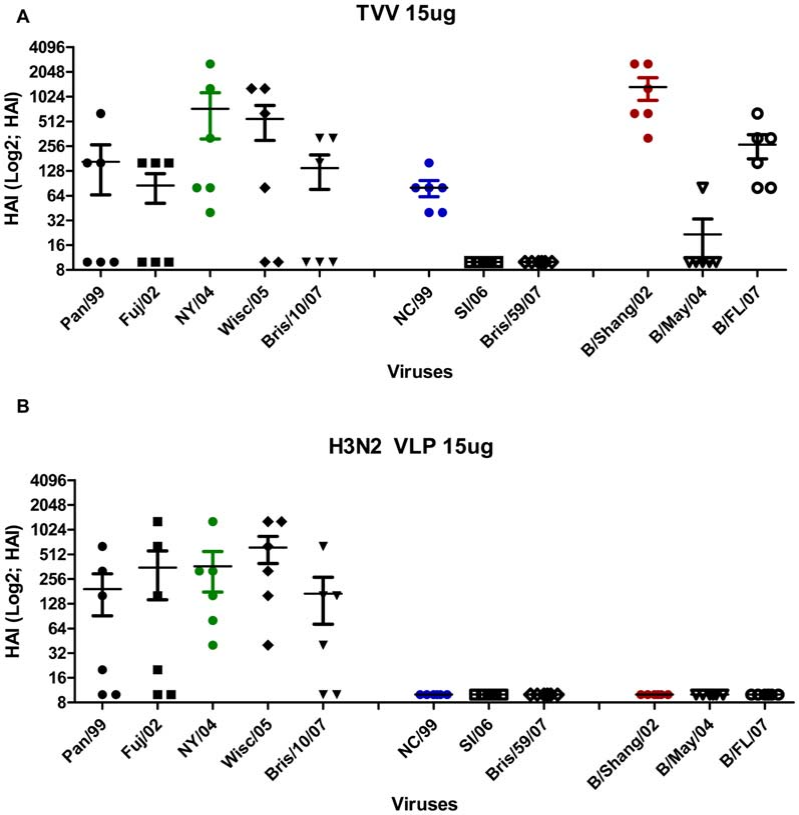
Trivalent vs. Monovalent comparison. HAI titers of ferrets vaccinated at the 15 µg dose with (A) trivalent VLP or (B) H3N2 VLP against a panel of influenza viruses as described in the legend to figure 6.

To confirm the effectiveness of these VLP vaccines, ferrets were vaccinated with 15 µg dose of the, monovalent H3N2 VLPs, TVV, or TIV. At week 3, all ferrets vaccinated with the TVV had high HAI titers against B/Shang/02, NC/99 and NY/04, albeit lower titers than against the influenza B virus (data not shown). The monovalent H3N2 VLP elicited similar titers after a single vaccination at week 3 as the TVV against NY/04. At week 5, all TVV vaccinated ferrets had HAI titers ≥1∶40 against all three viruses (NC/99, NY/04, B/Shang/02) in the vaccine (Fig. 6A). The HAI GMT against NC/99 was 1∶80, against NY/04 was 1∶727, and against B/Shang/02 was 1∶1280. Ferrets vaccinated with 15 µg dose of the monovalent H3N2 VLP had HAI titers against NY/04 (1∶367), but no cross-reactive HAI antibodies against NC/99 or B/Shang/02 (Fig. 6B). No ferrets vaccinated with lower doses of TVV (3 µg or 0.6 µg) had HAI titer ≥1∶40 against NC/99 or NY/04 (data not shown). The TVV elicited little or no cross-reactive HAI antibodies against the closely related SI/06 (H1N1) or B/May/04 viruses (Fig. 6A). However, both the TVV and the monovalent H3N2 VLP elicited HAI antibodies in 50–67% of ferrets against related H3N2 viruses, Pan/99, Fuj/02, Wisc/05, or Bris/10/07.

**Figure 7:**
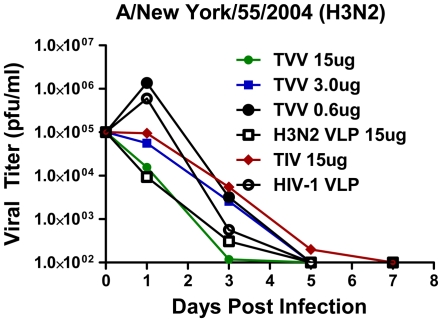
Protection of ferrets from influenza virus challenge. At week 5, ferrets vaccinated with TVV, H3N2 VLPs, TIV or HIV-1 VLPs were challenged intranasally with a 5×10^5^ pfu of NY/04 influenza virus. At days 1, 3, 5, and 7, ferret nasal washes were collected and virus titers were determined by plaque assay on MDCK cells. The data is plotted as the pfu/ml of virus in the nasal wash.

Ferrets were challenged intranasally with the H3N2 NY/04 virus (5×10^5^ pfu). HIV-1 VLP vaccinated ferrets showed clinical signs of infection (lethargy), a spike in temperature (∼2°C) in the first 24 hours post-challenge, and an increase in virus was detected in the nasal wash (8.25×10^5^ pfu/ml) (Fig. 7 and Table 2). Similar viral titers were observed in ferrets vaccinated with a 0.6 µg dose of TVV (1.57×10^6^ pfu/ml). However, there was a dose dependent decrease in viral titers that correlated with increasing doses of TVV. Ferrets vaccinated with 15 µg of TVV had similar viral titers in the nasal wash as ferrets vaccinated with 15 µg of the monovalent H3N2 VLP vaccine. In contrast, mice vaccinated with 15 µg of TIV had titers similar to the 15 µg of TVV. Viral titers dropped precipitously by day 3 post-challenge and no viruses were detected in the nasal wash after day 5 in any of the groups.

**Table 2:** Viral titers in vaccinated ferrets.

	Vaccine/Dose				
	TVV^a^ 15 mg	TVV 3 mg	TVV 0.6 mg	H3N2 VLP	HIV-1 VLP
Virus Titer	6.7×10e+3^b^	1.1×10e+5	3.2×10e+6	4.3×10e+3	5.1×10e+5
	1.3×10e+4	9.9×10e+4	2.0×10e+6	2.9×10e+3	1.9×10e+6
	1.7×10e+4	2.3×10e+4	5.9×10e+5	1.8×10e+4	4.5×10e+5
	3.2×10e+3	3.3×10e+4	1.2×10e+6	3.8×10e+3	3.9×10e+5
	4.8×10e+4	3.0×10e+4	8.0×10e+5	6.0×10e+3	1.1×10e+5
	5.3×10e+3	4.1×10e+4	4.8×10e+5	2.1×10e+4	2.3×10e+5

a Vaccine administered at weeks 0 and 3.

b pfu/ml on day 1 post-infection.

## Discussion

Immune responses elicited by a trivalent mixture of influenza virus-like particles expressing antigens from influenza A H1N1, H3N2, and influenza B viruses were shown to elicit immune responses in mice and ferrets as measured by serum HAI antibody titers and protection following viral challenge. For human influenza vaccinations, formalin-inactivated split virus that elicits HAI antibody titers in the range of 1∶40 are required to confer 50% protection against infection (PD_50_) [20]. HAI antibody titers ≥1∶40 are generally considered an immunological correlate of protection threshold beyond which it is unlikely that serious illness will occur [12], [19]. Following two vaccinations with the TVV, all mice and ferrets vaccinated with the highest doses of VLPs had HAI titers ≥1∶40 against the matched homologous viruses. Mice vaccinated with the each monovalent VLP elicited HAI titers against the homologous virus, whereas the TVV mixture elicited immune responses against all three influenza viruses (Fig. 2). There was a dose-dependent decline in the HAI titer in ferrets vaccinated with lower doses of TVV. There was no significant loss in HAI titer to a specific virus when the VLP was formulated in a trivalent mixture compared to the same monovalent VLP little immune interference or dominance of one VLP over others in the TVV.

HAI results from ferrets that were administered the same trivalent and monovalent VLP vaccines showed similar results as the mice against the homologous viruses to the vaccines (Fig. 6). However, only 50% of ferrets vaccinated with the TVV or the monovalent H3N2 VLP vaccine had cross-reactive HAI titers against a panel of H3N2 viruses isolated from 1999–2007. None of the ferrets had cross-reactivity against contemporary H1N1 viruses and only modest levels of cross-reactive HAI antibodies against influenza B viruses. Three of the ferrets had HAI titers ≥1∶256 and only sera from these three ferrets reacted against other H3N2 viruses. Mice and ferrets vaccinated with TIV had average HAI GMT greater than 1∶40, however, these titers were generally lower than those elicited by TVV.

Although HAI antibody titer is a generally accepted correlate of protection against influenza, these VLPs were effective at eliciting cellular responses, which may have contributed to protection or hastened the recovery of infected animals compared to TIV-vaccinated or mock vaccinated animals. Even though detection of cellular responses was low and sporadic prior to challenge, the induction of HA-specific, CD8^+^ IFN-γ secreting memory T cells was significantly higher in TVV-vaccinated mice compared to mice vaccinated with TIV (Fig. 3). To examine cell-mediated memory responses, we chose to collect splenocytes 6 days post-challenge to differentiate between primary and memory cellular responses. Even though CD8+ cytotoxic T cells (CTL) do not prevent infection, T cells provide some level of protection against influenza by promoting viral clearance and reducing the severity of symptoms [21]–[23]. Influenza-specific CTLs mostly target internal proteins, such as NP, [24]–[28] and can provide partial protection across heterologous strains [29]. VLPs evaluated in the study do not contain NP and therefore, only TIV-vaccinated mice elicited NP-specific CD8+ T cells over the background responses induced by primary viral infection. We cannot rule out that these NP-specific responses could have aided in the recovery of TIV-vaccinated mice. The VLPs used in this study induced CD8+ IFN-γ secreting cells that were directed at the HA that were significantly higher than the inactivated, split TIV indicating that CD8+ T cell responses against HA may be important in viral clearance. The elicitation of cross-reactive CD4+ and CD8+ T cell memory responses has been detected in healthy adults exposed to live influenza virus [29], [30]. While we did not directly measure CD4+ specific influenza responses, and the ELISPOT results presented in this study may be CD4^+^ T cell specific (Fig. 5), previous studies from our group demonstrated that our influenza VLP vaccines elicit a predominately IgG_2a_ and IgG_2b_ anti-HA antibodies, indicative of a T-helper type 1 biased CD4^+^ response [10]–[12].

We expected mice vaccinated with NC/99 VLPs to be protected against a NC/99 challenge. However, vaccinated mice lost weight and showed signs of morbidity. Mice are not a natural host for influenza infection and therefore, viruses need to be adapted to efficiently replicate in the mouse. During this process, mutations are introduced. Twelve amino acids were changed in the HA of the mouse-adapted NC/99 virus and the original human sequence. Since the vaccine contained the human sequences, the antibodies elicited recognized the HA sequence from the human virus, but not the mouse-adapted virus (Fig. 4). Four of these changes occurred in putative antigenic regions of HA. Future studies will be needed in order to determine which mutations are important for this phenomenon. We took advantage of the mouse-adapted virus to stimulate recall T cell responses elicited by the VLP vaccine.

Ferrets are generally considered a more relevant animal model for human influenza infection compared to mice [31]. While cross-reactive HAI antibodies were detected in mice, there was variability in homotypic cross-reactive HAI antibodies in ferrets, particularly at lower doses of VLP vaccine. Interestingly, there was a dose dependent correlation between the TVV dose and the viral titer isolated from the nasal wash of NY/04 infected ferrets (Fig. 7). In addition, viral titers were similar between ferrets vaccinated with the TVV and the monovalent H3N2 VLP vaccine administered at the same 15 µg dose, once again indicating that mixing the VLPs in a trivalent mix did not reduce immunogenicity. We cannot rule out that cellular immune responses played a role in the protection and fast decline in viral lung titers in TVV-vaccinated ferrets, since we were not able to measure cellular responses in ferrets. However, TVV-vaccinated ferrets did have antibodies that recognized NA that inhibited infection (data not shown). Humoral immunity elicited by neuraminidase (N1) can partially protect against H5N1 infection in a mammalian host [32] and anti-NA (N2) antibodies may have reduced the severity of disease associated with the antigen shift to H3N2 circulating viruses in 1968 [33]–[35]

An advantage of a VLP vaccine approach is a non-infectious mimicking particle with multiple viral antigens and epitopes that stimulate a diverse set of immune responses with less reactogenicity associated with a live-attenuated or whole-inactivated, split vaccine [10], [11]. In general, VLPs have the potential to activate both endogenous and exogenous antigen pathways leading to the presentation of viral peptides by MHC class I and class II molecules [36]. In this study, we showed that anti-HA specific CD8+ T cells were elicited by a seasonal influenza VLP vaccine indicating that a multi-epitope vaccine is more likely than an inactivated, split vaccine to generate a broad-based immune response. However, it is important to note that the CD8+ T cell epitopes studied in the mouse model have not been identified in humans. Particles, unlike single proteins, have the ability to bind and enter cells using appropriate surface receptors. These viral proteins can be processed and presented on MHC class I molecules, therefore promoting presentation to T-cells by professional antigen presenting cells. In addition, cell-free VLPs bound with antibodies could be taken up by phagocytic cells via Fc receptors, thus increasing MHC class II presentation [36]. Antigens expressed in their native three-dimensional conformational form can elicit more effective antibody responses compared to proteins in their non-native forms [37]. Many neutralizing antibodies directed against viruses are elicited against conformational epitopes only present in the native form of envelopes, and some epitopes are only exposed after binding to receptors during entry. Indeed, both HAI functional antibody was detected following vaccination with these VLP vaccines.

In humans, protection and clearance of influenza virus following infection is not only dependent upon virus virulence, but also the specific innate immunity, specific serum IgG antibody, and cell-mediated immunity of the individual infected. Our results show induction of complementary responses following vaccination with VLPs – antibodies that block HA binding and CD8+ T cell responses – that may likely contribute to protection from disease as demonstrated in the ferret model. In addition to a desirable immune response, trivalent preparations of VLPs benefit the public because they can easily be engineered and produced in a timely fashion, overcoming potential limitations of production of current egg-based influenza vaccines.
